# The relationship between infectious disease risk awareness and health literacy: A study among midwifery students

**DOI:** 10.12669/pjms.42.5.14469

**Published:** 2026-05

**Authors:** Sümeyye Ahi, Tuğba Çalışkan

**Affiliations:** 1 Dr. Sümeyye Ahi, PhD. Department of Midwifery, Faculty of Health Sciences, Kırşehir Ahi Evran University, Kırşehir, Türkiye; 2 Tuğba Çalişkan, MS Student. Department of Midwifery, Faculty of Health Sciences, Kırşehir Ahi Evran University, Kırşehir, Türkiye

**Keywords:** Health Literacy, Infectious Disease, Midwifery Students, Risk Awareness

## Abstract

**Objective:**

To determine the relationship between infectious disease risk awareness and health literacy levels among midwifery students and to identify factors associated with these variables.

**Methodology:**

This descriptive and correlational study was carried out between April 1, 2025 and June 1, 2025, with 231 midwifery students from a state university in central Türkiye. The sample size was calculated using Epi Info with a 95% confidence level, 50% prevalence, and 0.05 margin of error. Data were collected through classroom-based surveys using the Infectious Diseases Risk Awareness and Protection Scale (IDRAPS) and the Turkey Health Literacy Scale-32 (THLS-32). Descriptive statistics, independent samples t-test, One-Way ANOVA, Welch and post-hoc tests, and Pearson correlation analysis were performed.

**Results:**

The mean IDRAPS score was 149.7 ± 14.1, and the mean THLS-32 score was 33.5 ± 8.2, with higher scores indicating greater infectious disease risk awareness and higher health literacy, respectively. Fourth-year students had significantly higher IDRAPS scores, whereas third-year students showed significantly higher THLS-32 scores (p<0.05). Students reporting lower income than expenses had significantly higher scores on both scales (p<0.05). No significant differences were observed in scores according to parental education levels (p>0.05). A positive and moderate correlation was found between THLS-32 and IDRAPS scores (r=0.405; p<0.001).

**Conclusion:**

Midwifery students showed moderate health literacy and high infectious disease risk awareness. The positive association between these variables suggests that enhancing health literacy may support more effective infectious disease prevention behaviors among midwifery students.

## INTRODUCTION

With the emergence of the COVID-19 pandemic, infectious diseases have once again become a global public health priority, highlighting the crucial role of healthcare professionals in infection control.^1^ Due to direct patient contact in clinical practice, the risk of infection is markedly higher among healthcare workers and, in particular, among students preparing for healthcare professions.^2^ Midwifery students constitute a vulnerable group in this context, as they maintain close contact with mothers and newborns throughout their professional training. Therefore, strengthening their knowledge and awareness of infection control is of critical importance.^3^

Health literacy is defined as the ability of individuals to access, understand, evaluate, and apply health-related information, and it is considered one of the key determinants in the prevention of infectious diseases.^4^ Current literature indicates that low health literacy increases infection risk, reduces adherence to protective behaviors during outbreaks, and negatively impacts the use of healthcare services.^5^

Recent studies conducted among healthcare students have shown that, despite receiving professional education, levels of health literacy and awareness of infectious disease risks may vary depending on educational background, clinical exposure, and curriculum content. For instance, while some studies indicate that midwifery students may exhibit higher health literacy compared to other health science disciplines, others highlight the need for enhanced educational interventions regarding specific infectious agents such as Human Papillomavirus (HPV).^6^,^7^ Furthermore, most existing studies tend to examine health literacy and infectious disease awareness separately, rather than addressing the interaction between these two variables in an integrated manner. Understanding this relationship is critical, as midwifery students act as future advocates for public health, and inadequate health literacy may hinder their effectiveness in promoting essential preventive behaviors such as immunization and hygiene.^8^,^9^ This gap in the literature underscores the need for studies focusing specifically on midwifery students, who have unique responsibilities in maternal and newborn care, and highlights the importance of evaluating the relationship between infection awareness and health literacy in terms of professional competency.^10^

This study aimed to identify the relationship between infectious disease risk awareness, protective behaviors, and health literacy among midwifery students, thereby contributing to the development of educational programs and providing evidence-based recommendations for infection control practices.

## METHODOLOGY

This cross-sectional and correlational study was conducted between April 1 and June 1, 2025, at a state university in central Türkiye. The study population consisted of four hundred twelve midwifery students enrolled in the 2024–2025 academic year. The minimum sample size was calculated using Epi Info with a 95% confidence level, 50% expected prevalence, and 0.05 margin of error, yielding 199 students. The study was conducted using a convenience sampling method, and data were collected from volunteer students who agreed to participate. Data were collected from 232 students; one dataset containing an outlier was excluded, and analyses were conducted with 231 students.

### Ethical considerations:

Ethics approval was obtained from the institutional review board (Decision No: 2025/06/14; dated March 26, 2025). The study complied with the principles of the Helsinki Declaration. Institutional permission for data collection was also obtained. Written and verbal informed consent was obtained from all participants prior to data collection.

### Inclusion criteria:

- Students enrolled in the Midwifery Department of Kırşehir Ahi Evran University who voluntarily agreed to participate in the study were included.

### Exclusion criteria:

- Students who had any condition that could impair their ability to complete the questionnaire were excluded from the study.

### Data collection:

Data were obtained through in-class, face-to-face surveys administered before lessons began. Participation was voluntary, and informed consent was obtained.

### Data Collection Tools:

- *Personal Data Form:* This form was developed by researchers based on a review of the relevant literature and consists of 12 questions.^1^-^7^
- *Turkey Health Literacy Scale-32 (THLS-32):* THLS-32 evaluates health literacy across two domains (treatment and service; disease prevention and health promotion) and four information-processing stages. Scores are standardized between zero and fifty; higher scores indicate better health literacy. In this study, Cronbach’s alpha values were 0.89 and 0.90 for the subscales.^11^ According to the categorization defined in the literature, health literacy levels are classified as follows: 0–25 indicates insufficient health literacy, >25–33 indicates problematic/limited health literacy, >33–42 indicates adequate health literacy, and >42–50 indicates excellent health literacy.
- *Infectious Diseases Risk Awareness and Protection Scale (IDRAPS):* The scale assesses personal and societal protective awareness related to infectious diseases. It consists of 36 items, with total scores ranging from 36 to 180. Higher scores indicate greater awareness. In this study, Cronbach’s alpha was 0.94 for the total scale.^12^
- *Dependent and Independent Variables**:*** The dependent variables of the study were the students’ level of health literacy, infectious disease risk awareness, and level of protective behaviors. Sociodemographic characteristics such as age, year of study, and income level were evaluated as independent variables.

### Statistical Analysis:

Analyses were performed using IBM SPSS Statistics for Windows, Version 20.0 (IBM Corp., Armonk, NY, USA). Descriptive statistics (frequency, percentage, mean, standard deviation) were calculated. Skewness and kurtosis values indicated normal distribution. Independent samples t-test and One-Way ANOVA were used for comparisons. Welch and Tamhane’s T2 tests were applied when variances were not homogeneous; Bonferroni was used for homogeneous groups. Pearson correlation analysis examined the relationship between scale scores.

## RESULTS

The mean age of the participants was 21.3±2.8 years. Among the parents, 50.2% of mothers and 24.7% of fathers were at least primary school graduates. A total of 71.0% of the students were living in a dormitory, 86.1% reported having received training on infectious diseases, and 22.5% stated that there was a history of infectious disease among people in their close environment (Table-I). The mean total score of the IDRAPS was 149.7±14.1 (min: 36- max: 180). Among the subdimensions, the lowest scores were obtained in community protection awareness, while the highest scores were found in personal contact awareness. The mean score of the THLS-32 Health Literacy Scale was 33.5±8.2 (min: 0, max: 50). In this scale, the lowest scores were observed in the *evaluation of health information* subdimension, whereas the highest scores were found in the *application of health information* subdimension (Table-II). In comparisons by year of study, fourth-year students had significantly higher IDRAPS mean scores, while third-year students had significantly higher THLS-32 mean scores (p<0.05). No significant differences were found in IDRAPS scores by maternal education level, nor in THLS-32 scores by paternal education level (p>0.05). Students who reported having an income lower than their expenses had higher scores on both scales (p<0.05) (Table-III). A positive and moderate correlation was found between the THLS-32 and IDRAPS scores (r=0.405; p<0.001). As THLS-32 scores increased, IDRAPS scores also increased (Table-IV).

**Table-I:** Distribution of the Participants’ Demographic Characteristics

Variables		Mean±SD	Min	Max
Age		21.3±2.8	18	37
		n=231	%	
Year of Study	1st year	45	19.5	
	2nd year	84	36.4	
	3rd year	56	24.2	
	4th year	46	19.9	
Mother’s Educational Status	Primary school or below	116	50.2	
	Middle school graduate	63	27.3	
	High school graduate	36	15.6	
	University or above	16	6.9	
Father’s Educational Status	Primary school or below	57	24.7	
	Middle school graduate	73	31.6	
	High school graduate	57	24.7	
	University or above	44	19.0	
Employment in an Income-Generating Job	Yes	12	5.2	
	No	219	94.8	
Perceived Income Level	Income higher than expenses	41	17.7	
	Income equal to expenses	176	76.2	
	Income lower than expenses	14	6.1	
Primary Place of Residence	Dormitory	164	71.0	
	Home	67	29.0	
Presence of Chronic Illness	Yes	10	4.3	
	No	221	95.7	
Training Received on Infectious Diseases	Yes	199	86.1	
	No	32	13.9	
Source of Infectious Disease Training	During undergraduate education	129	55.8	
	Social media	28	12.1	
	Family/friends	23	10.0	
	Healthcare professionals	19	8.2	
Presence of Infectious Disease History in Close Contacts	Yes	52	22.5	
	No	179	77.5	
Encountering a Patient with an Infectious Disease During Internship	Yes	160	69.3	
	No	71	30.7	

**Table-II:** Distribution of the Participants’ IDRAPS and THLS-32 Scores

Scales (n = 231)	Mean ±SD	Min	Max
**IDRAPS Total Score**	**149.7±14.1**	**36**	**180**
***Subdimensions***			
Infectious Disease Risk Awareness	34.3±5.4	9	45
Personal Protection Awareness	34.8±3.6	21	40
Protective Behaviors	33.0±4.6	8	40
Handwashing Behaviors	13.4±1.6	3	15
Community Protection Awareness	15.8±2.3	4	20
Personal Contact Awareness	18.4±1.8	4	20
**THLS-32 Total Score**	**33.5±8.2**	**0**	**50**
*Subdimensions*			
**Treatment and Service Subdimension**	**34.5±8.3**	0	50
Accessing health information	36.2±8.7		
Understanding health information	36.1±9.4		
Evaluating health information	29.5±10.5		
Applying health information	36.3±9.8		
**Disease Prevention and Health Promotion Subdimension**	**32.5±9.3**	0	50
Accessing health information	35.1±9.5		
Understanding health information	35.2±9.1		
Evaluating health information	30.3±11.9		
Applying health information	29.1±12.0		
***Health Literacy Level According to the THLS-32 Scale***	***n=231***	***%***	
Inadequate HL	31	13.5	
Problematic–Limited HL	89	38.5	
Sufficient HL	74	32.0	
Excellent HL	37	16.0	

**Table III:** Comparison of Participants’ Characteristics with IDRAPS and THLS-32 Scores

Variables	n	IDRAPS Mean±SD	Test P	THLS-32 Mean±SD	Test P
***Year of Study***					
1st year	45	148.9±17.6	F/Welch= 2.972 p=0.035* 4-2**	35.0±8.0	F=6.851 P<0.000* 2-1,3**
2nd year	84	147.1±11.4		30.6±8.0	
3rd year	56	150.9±14.3		36.5±8.5	
4th year	46	153.8±13.7		33.5±6.7	
***Mother’s Educational Status***					
Primary school or below	116	148.1±12.9	F=1.528 p=0.208	33.7±8.2	F/Welch=2.038 p=0.118
Middle school graduate	63	152.7±15.2		34.5±7.0	
High school graduate	36	150.3±15.7		32.6±10.5	
University or above	16	148.5±13.9		30.3±8.2	
***Father’s Educational Status***					
Primary school or below	57	147.9±14.7	F=0.504 p=0.680	36.0±8.9	F/Welch=2.348 p=0.076
Middle school graduate	73	150.8±13.5		32.7±6.5	
High school graduate	57	150.3±15.4		33.0±9.5	
University or above	44	149.3±12.5		32.0±7.5	
***Employment in an Income-Generating Job***					
Yes	12	146.9±9.2	t=0.701	31.1±5.8	t=1.042
No	219	149.8±14.3	p=0.484	33.6±8.3	p=0.299
***Perceived Income Level***					
Income higher than expenses	41	155.2±2.0	F=6.005	37.9±1.2	F=8.052
Income equal to expenses	176	149.1±1.0	p=0.003*	32.6±1.0	p<0.000*
Income lower than expenses	14	141.4±4.3	1-2,3***	31.1±2.1	1-2,3***
***Presence of Chronic Illness***					
Yes	10	148.5±18.7	t=0.273	35.1±9.3	t=0.621
No	221	149.7±13.9	p=0.785	33.4±8.2	p=0.535
***Training Received on Infectious Diseases***					
Yes	199	150.0±13.8	t=0.922	33.6±8.0	t=0.747
No	32	147.6±15.6	p=0.357	32.5±9.4	p=0.456
***Source of Infectious Disease Training***					
During undergraduate education	129	149.8±14.7	F=0.340 p=0.797	34.6±7.8	F=1.914 p=0.129
Social media	28	150.5±10.2		31.6±6.4	
Family/friends	23	148.4±14.9		31.7±9.1	
Healthcare professionals	19	152.6±11.3			
***Presence of Infectious Disease History in Close Contacts***					
Yes	52	149.6±14.0	t=0.078	33.7±7.6	t=0.218
No	179	149.7±14.1	p=0.938	33.4±8.4	p=0.828
***Encountering a Patient with an Infectious Disease During Internship***					
Yes	160	150.8±13.1	t=1.722	34.1±8.3	t=1.659
No	71	147.3±15.8	p=0.086	32.1±7.8	p=0.098
***Health Literacy Levels***					
Inadequate^1^	31	144.812.0	F=17.446 <0.000* 4-1,2,3***
Problematic–Limited^2^	89	144.611.7	
Sufficient^3^	74	152.014.4	
Excellent^4^	37	161.412.4	

F= OneWay ANOVA, t= Independent samples t-test, *p<0.05; **Post-Hoc test, Tamhane’s

*** Post-Hoc test, Dunn Bonferroni.

**Table IV:** Relationship Between IDRAPS and THLS-32 Scores.

Scale		IDRAPS
THLS-32	Pearson Correlation (r)	0.405
	*p*	<0.000*
	*n*	231

* p<0.05.

## DISCUSSION

This study examined the relationship between midwifery students’ health literacy levels and their awareness of infectious disease risks, revealing a positive and moderate association between the two variables. This indicates that higher health literacy enhances risk perception and engagement in protective behaviors. Our findings align with existing literature showing that students with higher health literacy demonstrate greater awareness and protective behavior regarding infectious diseases, including COVID-19.^13^ Similar effects of health literacy on protective behaviors have also been reported in different populations.^10^

In our study, midwifery students demonstrated generally high levels of infectious disease risk awareness. However, previous studies have reported lower levels among university students.^14^,^15^ This difference should be interpreted with caution, as the characteristics of the study samples vary across studies. In the study by Erdoğan and Duru, the sample consisted of university students from various non-health and social science-related departments, whereas Baykal and Akar included students from health sciences programs. Therefore, direct comparisons between studies may be limited due to differences in educational background and exposure to health-related knowledge. The higher awareness observed in our sample may be explained by the fact that all participants were midwifery students, a group receiving specialized education in health. This suggests that students in health-related programs may have a more developed understanding of infectious disease risks compared to students from non-health disciplines. In addition, the emphasis on infection control in the curriculum and the clinical experience gained during internships may have contributed to the higher awareness levels observed in this study.

When the relationship between health literacy and infectious disease risk awareness was examined, a positive and moderate correlation was found between THLS-32 and IDRAPS scores. This indicates that as health literacy increases, risk perception and protective behaviors also improve. The literature supports this finding: a positive association between health literacy and COVID-19 awareness has been reported.^13^ Similar findings have been reported in studies published in Pakistan-based journals, where higher levels of knowledge and awareness were associated with better preventive practices among healthcare professionals.^16^ Orhan et al. (2025) showed that students in health-related fields have higher protective behavior scores, while Sarimehmet et al. (2024) found that health literacy increases risk perception and protective behaviors among prisoners.^10^,^17^ These findings reinforce the role of health literacy as a key determinant in shaping individuals’ responses to infectious disease risks across different populations. These results collectively show that our findings are consistent with the existing literature.

In the comparison by class level, fourth-year students had higher IDRAPS scores, while third-year students had higher THLS-32 scores. This finding differs from previous studies examining class-level differences. Keten and Yörük found no consistent pattern between class levels, with the highest scores in second-year and the lowest in fourth-year students.^18^ These variations may be related to differences in curriculum structure, timing of clinical practice, and students’ field experience. In particular, variations in the timing and intensity of clinical exposure across academic years may explain these inconsistencies. In midwifery programs, the intensive internship period in the third year may expose students more directly to infectious disease risks, contributing to increased awareness during that stage.

Although parental education is often considered an important factor in shaping students’ health-related awareness,^19^ our study found no significant differences in health literacy scores based on parental education level. In contrast, a study conducted in Nepal reported that higher parental education increased students’ health literacy,^20^ and similar findings were also noted in a systematic review.^19^ This discrepancy may be explained by the relatively homogeneous educational environment of university students, where formal education may play a more dominant role than family background. These differences suggest that the influence of parental education may vary by context and may be more limited among university students, particularly those in health-related fields.

This study contributes new insight by examining health literacy and infectious disease risk awareness together in a midwifery student population. Unlike many previous studies that address these variables separately, this study provides evidence of their combined effect, thereby contributing to a more comprehensive understanding of student competencies in infection control. The findings underscore the importance of improving health literacy to enhance infection control and support safe clinical practice. Overall, the results highlight the need for structured educational strategies within midwifery curricula to strengthen health literacy and better prepare students for infection-related risks.

### Strengths of the study:

It examines health literacy and infectious disease risk awareness together, addressing a gap in the literature, and focuses specifically on midwifery students. The use of validated measurement tools also strengthens the findings. However, the study is limited to a single institution, and future studies including different populations and longitudinal designs are recommended.

### Limitations:

This study was conducted with midwifery students from a single university; therefore, the findings cannot be generalized to other institutions. The use of self-reported data carries the risk of social desirability bias. In addition, because the data were collected during a specific period, temporal changes in students’ knowledge and awareness levels were not assessed.

## CONCLUSION

Midwifery students demonstrated high infectious disease risk awareness and moderate health literacy levels. A positive relationship between the two variables indicates that health literacy may play an important role in students’ recognition of infectious disease risks. These findings highlight the need to strengthen health literacy-focused educational strategies within midwifery curricula to support safer clinical practice.

### Authors’ Contribution:

**SA:** Literature review, Conceptualization, Design, Data collection, Data analysis and translation, Critical review and Manuscript writing

**TC:** Literature review, Conceptualization, Design, Data collection and Manuscript writing.

All authors have approved the final version of the manuscript and are responsible for all aspects of the work.

## References

[1] World Health Organization (2022). Infection prevention and control in healthcare.

[2] Centers for Disease Control and Prevention (2021). Infection prevention and control recommendations.

[3] International Confederation of Midwives (2020). Midwives'role in infection prevention and control.

[4] Okan O, Messer M, Levin-Zamir D, Paakkari L, Sørensen K (2023). Health literacy as a social vaccine in the COVID-19 pandemic. Health Promot Int.

[5] Paakkari L, Okan O (2020). COVID-19: health literacy is an underestimated problem. Lancet.

[6] Güllü A, Aloğlu N (2023). Relationship between mindfulness and HPV knowledge levels among midwifery and nursing students. J Health Sci Inst (Istanbul Univ).

[7] Yorulmaz DS, Sezer HK (2021). Health literacy and associated factors among nursing students receiving education in first aid and emergency services. Inonu Univ J Health Serv.

[8] Akbal E, Gökler ME (2020). A reality revealed during the COVID-19 pandemic: health literacy. ESTUDAM Public Health J.

[9] Çelebi E (2022). Protective behaviors, risk perceptions, and COVID-19 vaccine acceptance of midwifery students regarding COVID-19 infection. KTO Karatay Univ J Health Sci.

[10] Orhan Z, Bilal Ş, Doğaner A, Kayış A, Doğan S (2025). Investigation of health literacy levels among university students regarding risk awareness and protection in infectious diseases. Ethiop J Health Dev.

[11] Okyay P, Abacıgil F, Harlak H (2016). Turkish Health Literacy Scales: Reliability and Validity Study. Ankara:Republic of Türkiye Ministry of Health Publications.

[12] Ener D, Seyfeli Y, Cetinkaya F (2022). A Scale Development and Validation Study: Communicable Diseases Risk Awareness and Protection Scale. Istanbul Med J.

[13] Akgül E, Tanrıkulu F, Dikmen Y (2023). Health literacy levels and COVID-19 awareness among university students in health sciences. Etkili Hemşirelik Derg.

[14] Erdoğan EG, Duru P (2025). Communicable disease risk awareness and prevention: a study on university students in the context of social support and disaster risk. Public Health Nurs.

[15] Baykal D, Akar EN (2025). Awareness of communicable disease risks among health sciences students. Eurasian J Health Sci.

[16] Ahmed N, Shakoor M, Vohra F, Abduljabbar T, Mariam Q, Rehman MA (2020). Knowledge, awareness and practice of health care professionals amid SARS-CoV-2, coronavirus disease outbreak. Pak J Med Sci.

[17] Sarimehmet D, Sarimehmet YK, Candas Altinbas B, Ardic C (2024). Relationship between infectious disease risk awareness, prevention behaviours, and health literacy among prisoners. Public Health.

[18] Keten S, Yörük G (2025). Risk awareness, protective behaviors, and attitudes toward infectious diseases among health sciences students: a descriptive cross-sectional study. Turk Klin Health Sci.

[19] Budhathoki SS, Pokharel PK, Jha N, Moselen E, Dixon R, Bhattachan M (2019). Health literacy of future healthcare professionals: a cross-sectional study among health sciences university students in Nepal. Int Health.

[20] Kühn L, Bachert P, Hildebrand C, Kunkel J, Reitermayer J, Wäsche H (2021). Health literacy among university students: a systematic review of cross-sectional studies. Front Public Health.

